# The Impact of Canopy Position on the Biochemical, Nutritional, and Nutraceutical Quality Characteristics of Grapefruit Cultivars During Fruit Growth and Development

**DOI:** 10.3390/plants15111750

**Published:** 2026-06-04

**Authors:** Meryam Manzoor, Muhammad Akbar Anjum

**Affiliations:** Department of Horticulture, Bahauddin Zakariya University, Multan 60800, Pakistan; meryam.manzoor@gmail.com

**Keywords:** nutraceutical quality, antioxidant capacity, biochemical constituents, canopy microclimate, harvesting time

## Abstract

Citrus fruit quality traits are governed by canopy position, harvest maturity, and cultivar, influencing nutritional and nutraceutical profiles. Grapefruit (*Citrus paradisi* Macf.) is recognized for its antioxidant-rich, health-promoting properties; however, limited information exists on how canopy microclimate interacts with developmental stages to affect nutritional-quality attributes. In a two-year study, four grapefruit cultivars (Rio Red, Star Ruby, Shamber, and Flame) were evaluated to determine the effects of canopy position (outer vs. inner) and harvest time on biochemical characteristics, antioxidant potential, and pigment accumulation under subtropical conditions in Pakistan. Fruits were collected monthly from August to December (6–10 months after anthesis; MAA). The results demonstrated that canopy position and harvest time had pronounced effects on fruit quality. Outer canopy fruits presented higher total soluble solids, ripening index, sugars, pigment accumulation, and antioxidant compounds across development stages. Fruit quality improved with maturity, and peaked in mid-December with maximal total soluble solids, ripening index, sugars, and pigment content accumulation. Overall, all the cultivars showed similar profiles in the change in fruit quality traits during growth and development. Rio Red and Star Ruby outperformed Shamber and Flame across most traits, highlighting cultivar-specific differences in metabolite accumulation. Together, canopy microclimate, harvest maturity, and cultivar are key determinants of bioactive–nutritional quality traits in grapefruit. In practice, managing canopy exposure and harvest windows with cultivar choice enhances health-promoting metabolites and nutritional quality, with added gains in commercial value and tree productivity.

## 1. Introduction

Citrus fruits are recognized globally not only for their sensory appeal but also for their nutritional aspects and health benefits. Citrus fruits have excellent aroma, flavor, and taste characteristics, with numerous health benefits [1]. The accumulation of different kinds of sugars, organic acids, pigments, antioxidants, and other quality-determining compounds in citrus fruits is influenced by cultural practices, cultivar types, and rootstock selection [2,3]. Given the nutritional and medicinal value of grapefruit, which is rich in ascorbic acid, vitamin A, and lycopene, fruit quality is essential for market acceptance and for meeting consumer expectations regarding overall eating quality [2,3]. Although the cultivation of grapefruit is widespread worldwide because of its greater yield, excellent flesh color, and higher nutritional value, it remains limited in Pakistan and could be improved through the introduction of superior cultivars and varietal diversity [4].

Fruit bioactive–nutritional quality attributes are influenced by climatic conditions, cultivar, canopy position, maturity stage, and other preharvest factors [5,6]. Canopy-driven microclimate variation plays a critical role in determining final fruit quality. The canopy microclimate of a tree depends on irradiance and sun exposure [7]. The amount of light penetrating the canopy is greater at the outside and results in a higher rate of assimilation of the leaves [8]. Moreover, canopy research in peaches has demonstrated direct correlations between light intensity in canopy areas and the ratio of fruit sugars to acid and color development [9]. Therefore, understanding how the canopy microclimate impacts fruit biochemical and quality-related physicochemical attributes is essential for improving overall fruit quality.

Fruit harvesting at the proper maturity stage is essential for optimizing fruit yield and quality. Different maturity indices (fruit color, weight and size, juice content, and organic acid conversion into sugars) determine the precision of the harvesting time of fruit crops [3,6]. The variation in maturity indices among different cultivars could be due to their diverse genetic makeup, environmental circumstances, and soil conditions [6]. Postharvest biochemical changes in citrus fruits occur at minimal levels because of their nonclimacteric nature, so harvesting at proper eating quality development is essential [10]. Therefore, fruits were sampled at different months to determine which indices are most suitable for defining the proper stage of fruit development/maturity. Harvest stages (August–December) were expressed in months after anthesis (MAA) to enable comparisons across citrus-growing regions with different flowering calendars.

Cultivar variation has a considerable effect on fruit biochemical composition, health promoting metabolites accumulation, and sensory quality traits because genetic variation controls the production of pigments, sugar–acid ratio, and antioxidant capacity [11]. The production of grapefruit is not very strong in Pakistan, with Shamber as the primary commercial cultivar. Therefore, Rio Red, Star Ruby, and Flame were added to this study to increase varietal diversity and find high-quality substitutes known internationally for their high pigmentation, high antioxidant capacity, and good taste.

Given the increasing demand for nutritionally balanced and antioxidant-containing citrus fruits, it is necessary to assess the effects of canopy microclimate, harvest maturity, and cultivar genetics on the biochemical composition, health-promoting metabolites, and nutritional value of grapefruit. However, few studies have explored how these factors interact to regulate fruit quality traits under subtropical conditions. Therefore, this two-year study was designed to evaluate the influence of canopy position (outer vs. inner) and harvest maturity (August–December) on the biochemical composition, pigment development, and antioxidant potential of four grapefruit cultivars. These findings are expected to help optimize canopy management, cultivar choice, and harvest timing to obtain nutritional quality and increase the commercial potential of grapefruit for both fresh consumption and juice processing.

## 2. Results

The data for each harvesting stage were analyzed separately using two-way ANOVA (cultivars × canopy positions). The mean values for canopy positions and the cultivars are represented in the Tables, while the interaction effects between cultivars × canopy positions are shown graphically in the Figures.

### 2.1. Impact of Canopy Positions on Juice Characteristics of Different Grapefruit Cultivars Harvested at Different Development Stages (August–December; 6 MAA–10 MAA)

Canopy position significantly influenced juice quality attributes of the grapefruit cultivars across the harvesting stages (Table 1). Fruits harvested from the outer canopy generally exhibited higher TSSs than those from the inner canopy, including August (6.64 vs. 5.89 °Brix), September (7.67 vs. 6.89 °Brix), October (7.98 vs. 7.20 °Brix), November (8.11 vs. 7.33 °Brix), and December (8.13 vs. 7.35 °Brix). In contrast, juice acidity remained higher in fruits collected from the inner canopy, with values in August (3.27 vs. 2.58%), September (2.76 vs. 2.08%), October (1.67 vs. 1.07%), November (1.18 vs. 0.95%), and December (1.15 vs. 0.90%) for inner and outer canopy positions, respectively. Likewise, the ripening index was consistently higher in fruits harvested from the outer canopy than inner canopy fruits, increasing from August (2.61 vs. 1.81) to December (9.21 vs. 6.50) (Table 1).

A significant variation among the cultivars was also observed for juice quality traits (Table 2). In August, the highest TSSs (6.90 °Brix) were noted in Rio Red, followed by Shamber (6.58 °Brix) and Flame (6.18 °Brix), whereas Star Ruby exhibited the lowest value (5.41 °Brix). In September and October, Rio Red exhibited the highest TSSs (7.94 and 8.25 °Brix, respectively), while Star Ruby showed the lowest values (6.41 and 6.72 °Brix, respectively). A similar pattern was observed during November and December, where Rio Red maintained the highest TSSs values (8.38 and 8.42 °Brix, respectively), whereas Star Ruby showed the lowest values (6.85 and 6.87 °Brix, respectively) (Table 2).

Regarding fruit juice acidity, cultivar differences were non-significant during August, September, and October. However, significant variation among the cultivars was seen during November and December, where Star Ruby recorded the highest acidity (1.15 and 1.13%, respectively), while Rio Red showed comparatively lower acidity levels during the same stages (1.01 and 0.92%, respectively) (Table 2). Similarly, Rio Red exhibited the highest ripening index throughout the harvesting period, with values increasing from August to December (2.62, 3.76, 6.66, 8.37, and 9.53). In contrast, Star Ruby recorded comparatively lower ripening index values, with August–December values increasing (1.84, 2.63, 5.12, 6.16, and 6.20) (Table 2).

**Figure 1:**
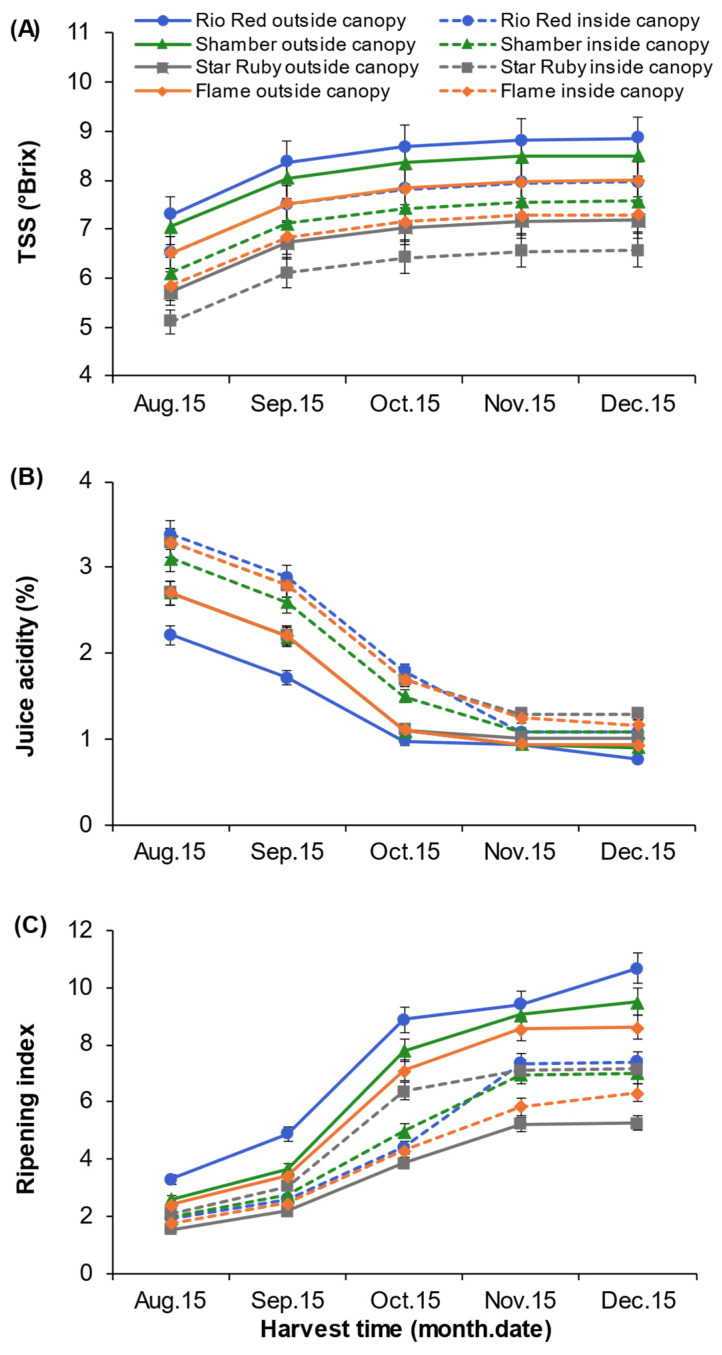
Changes in TSSs (**A**), juice acidity (**B**), and ripening index (**C**) of the grapefruit cultivars, as affected by cultivar and canopy positions at different harvest times (stages). The error bars represent the standard errors of the means (*n* = 3). The data for each harvesting stage were analyzed separately using two-way ANOVA (cultivar × canopy position), and the interaction means are presented graphically to show the stage-wise response of the cultivars across canopy positions during fruit development. Significance levels indicated as *p* < 0.01 (**), *p* < 0.05 (*), and NS = non-significant. CP × C represents canopy positions × cultivars. TSSs CP × C: August–December^NS^. Juice acidity CP × C: August–September *, October–December^NS^. Ripening index CP × C: August *, September *, October *, November^NS^, and December *.

The canopy position × cultivar interaction varied across the harvesting stages (Figure 1). For TSSs, the interaction was non-significant from August to December (Figure 1A). Regarding juice acidity, a significant interaction was observed in August and September, where fruits from the inner canopy showed higher acidity across the cultivars, while the lowest acidity was observed in Rio Red fruits from the outer canopy; the remaining stages were non-significant (Figure 1B). In contrast, the interaction for the ripening index was significant during August, September, October, and December, and became non-significant during November. Across these stages, the highest ripening index was observed in Rio Red fruits harvested from the outer canopy, while the lowest values were recorded in Star Ruby fruits from the inner canopy (Figure 1C).

### 2.2. Impact of Canopy Positions on Sugar Content in Different Grapefruit Cultivars Harvested at Different Development Stages (August–December; 6 MAA–10 MAA)

Canopy position showed a significant effect on sugar accumulation in the grapefruit cultivars across the harvesting stages (Table 1). Fruits harvested from the outer canopy generally exhibited higher reducing sugar than those from the inner canopy, including August (3.16 vs. 3.12%), September (3.80 vs. 3.73%), October (4.50 vs. 4.42%), November (4.94 vs. 4.89%), and December (5.24 vs. 5.18%). For nonreducing sugars, canopy position differences remained non-significant during August (1.47 vs. 1.46%). However, fruits harvested from the outer canopy showed comparatively higher nonreducing sugar levels during the remaining stages, including September (1.69 vs. 1.64%), October (2.10 vs. 2.07%), November (2.81 vs. 2.76%), and December (2.87 vs. 2.82%). Similarly, for total sugar, fruits harvested from the outer canopy generally showed higher values than those from the inner canopy, including August (4.63 vs. 4.58%), October (6.60 vs. 6.49%), November (7.76 vs. 7.65%), and December (8.11 vs. 8.00%). In contrast, the values during September remained not significantly different between the outer and inner canopy positions (5.45 vs. 5.43%) (Table 1).

Regarding the cultivars, a significant variation among the cultivars was observed for sugar composition (Table 2). For reducing sugar, Rio Red recorded the highest values across all harvesting stages, including August–December harvest times (4.00, 4.46, 5.44, 5.87, and 6.17%), whereas Star Ruby exhibited comparatively lower values during the same stages (2.46, 2.94, 3.62, 4.33, and 4.44%). For nonreducing sugars, Star Ruby recorded the highest value in August (1.52%), followed by Shamber (1.50%), while Flame had the lowest value (1.38%). During September and October, Rio Red showed the highest nonreducing sugar levels (1.99% and 2.22%, respectively), while Star Ruby and Shamber had comparatively lower values in September (both 1.52%), and Star Ruby showed the lowest in October (1.95%). However, in November and December, Shamber recorded the highest nonreducing sugar levels (3.02% and 3.11%, respectively), while Star Ruby displayed relatively lower values (2.44% and 2.69%, respectively) during the same months (Table 2). Similarly, greater total sugar content was noted in Rio Red throughout the harvesting period, with values increasing from August to December (5.47, 6.46, 7.67, 8.77, and 8.99%). In contrast, Star Ruby showed comparatively lower total sugar levels during the same months (3.98, 4.47, 5.57, 6.77, and 7.14%), while Shamber and Flame recorded intermediate values across the harvesting stages (Table 2).

**Figure 2:**
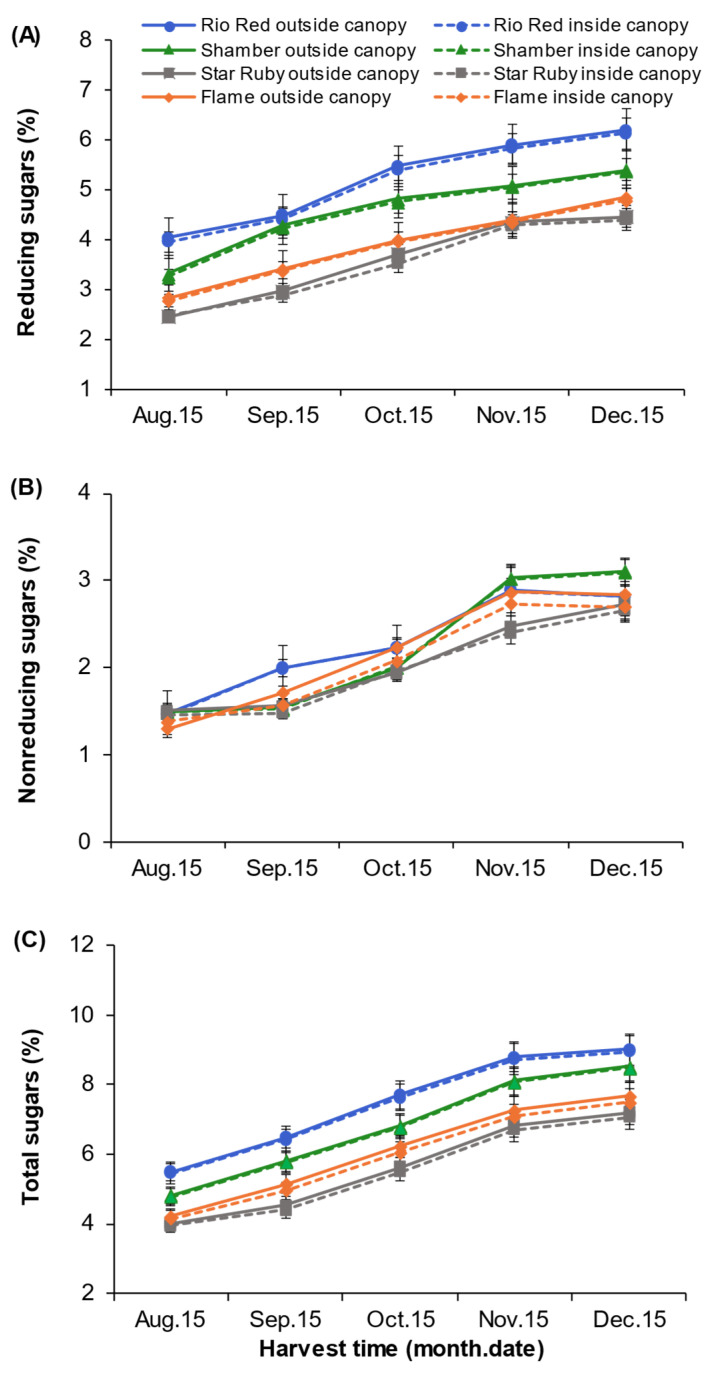
Changes in reducing sugars (**A**), nonreducing sugars (**B**), and total sugars (**C**) in the grapefruit cultivars, as affected by the cultivar and canopy positions at different harvest times (stages). The error bars represent standard errors of the means (*n* = 3). The data for each harvesting stage were analyzed separately using two-way ANOVA (cultivar × canopy position), and the interaction means are presented graphically to show the stage-wise response of the cultivars across canopy positions during fruit development. Significance levels indicated as *p* < 0.01 (**), *p* < 0.05 (*), and NS = non-significant. CP × C represents canopy positions × cultivars. Reducing sugars CP × C: August and September^NS^, October *, November, and December^NS^. Nonreducing sugars CP × C: August^NS^, September–December *. Total sugars CP × C: August^NS^, September–December *.

The canopy position × cultivar interaction varied across the harvesting stages (Figure 2). For reducing sugars, the interaction was non-significant from August to December, and was significant only during October (Figure 2A). In October, higher reducing sugar levels were observed in Rio Red fruits from both canopy positions, whereas the lowest values were recorded in Star Ruby fruits from the inner canopy. For nonreducing sugars, the interaction remained non-significant during August and was significant from September to December (Figure 2B). Across these stages, higher nonreducing sugar levels were generally observed in Rio Red and Shamber fruits from both canopy positions, whereas the lowest values were consistently recorded in Star Ruby fruits, particularly from the inner canopy. A similar pattern was observed for total sugars, where the highest values were consistently recorded in Rio Red fruits from the outer canopy, while the lowest values were observed in Star Ruby fruits from the inner canopy (Figure 2C).

### 2.3. Impact of Canopy Positions on Bioactive Compounds in Different Grapefruit Cultivars Harvested at Different Development Stages (August–December; 6 MAA–10 MAA)

Bioactive compounds, including ascorbic acid, total phenolics, flavonoids, and antioxidant capacity, were evaluated together as indicators of the antioxidant potential, nutritional, and nutraceutical quality of grapefruit juice.

Canopy position significantly influenced the bioactive composition in the grapefruit cultivars across the harvesting stages (Table 3). Fruits harvested from the outer canopy generally exhibited higher ascorbic acid content than those from the inner canopy, including August (59.62 vs. 57.10 mg 100 mL^−1^), September (57.62 vs. 55.10 mg 100 mL^−1^), October (57.62 vs. 55.10 mg 100 mL^−1^), November (55.12 vs. 52.60 mg 100 mL^−1^), and December (54.87 vs. 52.35 mg 100 mL^−1^). A similar trend was seen for total phenolic content, where fruits harvested from the outer canopy exhibited higher values compared with the inner canopy, including August (84.63 vs. 82.69 mg GAE 100 mL^−1^), September (82.62 vs. 80.68 mg GAE 100 mL^−1^), October (80.14 vs. 78.20 mg GAE 100 mL^−1^), November (78.60 vs. 76.66 mg GAE 100 mL^−1^), and December (78.27 vs. 76.33 mg GAE 100 mL^−1^). Likewise, antioxidant capacity remained higher in fruits collected from the outer canopy, with values of August (36.36 vs. 34.38 mM Trolox 100 mL^−1^), September (34.11 vs. 32.15 mM Trolox 100 mL^−1^), October (30.49 vs. 28.54 mM Trolox 100 mL^−1^), November (28.37 vs. 26.41 mM Trolox 100 mL^−1^), and December (27.47 vs. 25.51 mM Trolox 100 mL^−1^) for outer and inner canopy positions, respectively. For flavonoids, fruits harvested from the outer canopy also showed higher values of flavonoids than the inner canopy during most harvesting stages, including August (15.57 vs. 13.59 mg QE 100 mL^−1^), October (26.61 vs. 24.62 mg QE 100 mL^−1^), November (29.81 vs. 27.83 mg QE 100 mL^−1^), and December (31.33 vs. 29.34 mg QE 100 mL^−1^). However, during September, flavonoid content did not significantly change among canopy positions (20.33 vs. 20.42 mg QE 100 mL^−1^) (Table 3).

Notably, significant variation among the cultivars was also observed for bioactive compounds across the harvesting stages (Table 4). For ascorbic acid, Rio Red and Shamber generally recorded higher values compared with the other cultivars throughout the harvesting period, with August–December values of 60.00, 58.25, 58.00, 55.50, and 55.25 mg 100 mL^−1^ and 60.46, 58.46, 58.25, 55.96, and 55.71 mg 100 mL^−1^, respectively. In contrast, comparatively lower values were noted for Star Ruby during the same stages, with August–December values of 55.00, 53.25, 53.00, 50.50, and 50.25 mg 100 mL^−1^. For total phenolic content, Rio Red and Star Ruby showed higher values during August (86.45 and 86.82 mg GAE 100 mL^−1^, respectively) than the other cultivars. During the remaining harvesting stages, consistently higher phenolic content was noted in Star Ruby, with September–December values of 85.07, 83.17, 81.67, and 81.61 mg GAE 100 mL^−1^. In contrast, Shamber recorded comparatively lower phenolic levels across the harvesting stages, including August–December values of 79.16, 77.17, 74.62, 73.46, and 72.50 mg GAE 100 mL^−1^ (Table 4). Regarding antioxidant capacity, Star Ruby consistently displayed the highest values across the harvesting stages, including August–December (38.12, 35.82, 32.82, 30.32, and 29.73 mM Trolox 100 mL^−1^). In contrast, Shamber exhibited comparatively lower antioxidant capacity during the same stages, with values of 32.13, 30.00, 25.90, 23.90, and 22.90 mM Trolox 100 mL^−1^ (Table 4).

A similar cultivar pattern to antioxidant capacity was noted for flavonoids. Star Ruby consistently recorded the highest values throughout the harvesting stages, with August–December values of 15.68, 21.83, 27.98, 31.83, and 32.88 mg QE 100 mL^−1^. In contrast, Shamber exhibited comparatively lower flavonoid levels during the same stages, with values of 13.24, 18.24, 22.04, 25.02, and 27.02 mg QE 100 mL^−1^ (Table 4).

**Figure 3:**
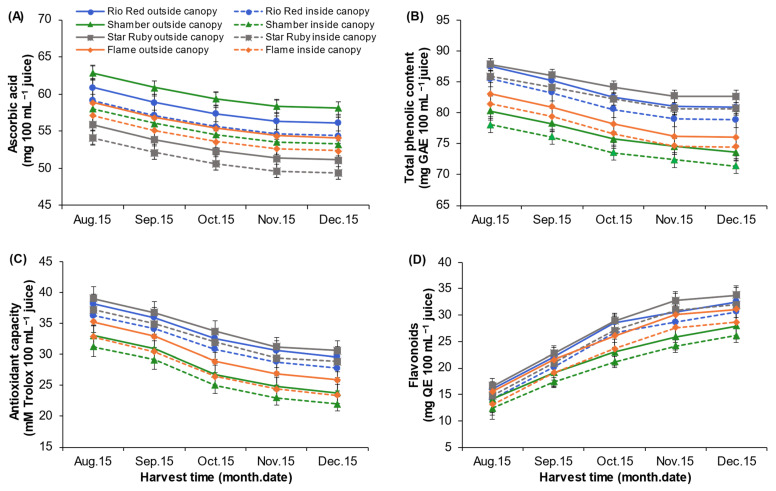
Changes in ascorbic acid (**A**), total phenolic content (**B**), antioxidant capacity (**C**), and flavonoids (**D**) in the grapefruit cultivars, as affected by the cultivar and canopy positions at different harvest times (stages). The error bars represent the standard errors of the means (*n* = 3). The data for each harvesting stage were analyzed separately using two-way ANOVA (cultivar × canopy position), and the interaction means are presented graphically to show the stage-wise response of the cultivars across canopy positions during fruit development. Significance levels indicated as *p* < 0.01 (**), *p* < 0.05 (*), and NS = non-significant. CP × C represents canopy positions × cultivars. Ascorbic acid CP × C: August-December *. Total phenolic content CP × C: August–December^NS^. Antioxidant capacity CP × C: August-December^NS^. Flavonoids CP × C: August–December^NS^.

The canopy position × cultivar interaction was significant only for ascorbic acid across all harvesting stages (August–December) (Figure 3A). Across the stages, the highest ascorbic acid levels were noted in Shamber fruits from the outer canopy, whereas the lowest values were observed in Star Ruby fruits from the inner canopy. In contrast, the interaction remained non-significant for total phenolic content, antioxidant capacity, and flavonoids across all harvesting stages (Figure 3B–D).

### 2.4. Impact of Canopy Positions on Pigment Content in Different Grapefruit Cultivars Harvested at Different Development Stages (August–December; 6 MAA–10 MAA)

Color-related pigments, including carotenoids and anthocyanins, were evaluated to assess variation among the cultivars and changes in grapefruit pigmentation during fruit development.

Canopy position influenced pigment accumulation in grapefruit across the harvesting stages; although the differences among the mean values were minimal, they were statistically significant (Table 3). Fruits harvested from the outer canopy generally recorded slightly but significantly higher carotenoid content than those from the inner canopy, including August (2.09 vs. 2.06 mg 100 mL^−1^), September (2.40 vs. 2.36 mg 100 mL^−1^), October (2.76 vs. 2.73 mg 100 mL^−1^), November (3.15 vs. 3.12 mg 100 mL^−1^), and December (3.35 vs. 3.32 mg 100 mL^−1^). Similarly, anthocyanin content remained significantly higher in fruits collected from the outer canopy, with values in August (0.29 vs. 0.27 mg 100 mL^−1^), September (0.46 vs. 0.43 mg 100 mL^−1^), October (0.62 vs. 0.60 mg 100 mL^−1^), November (0.77 vs. 0.74 mg 100 mL^−1^), and December (0.85 vs. 0.83 mg 100 mL^−1^) for outer and inner canopy positions (Table 3).

Among the cultivars, significant variations were noted for pigment composition across the harvesting stages (Table 4). For carotenoids, Star Ruby consistently recorded the highest values throughout the harvesting period, with August–December values of 3.70, 4.10, 4.52, 5.04, and 5.24 mg 100 mL^−1^. In contrast, Shamber exhibited comparatively lower carotenoid levels during the same stages, including 1.01, 1.31, 1.52, 1.73, and 1.83 mg 100 mL^−1^ (Table 4). A similar cultivar pattern was observed for anthocyanins, where Star Ruby consistently recorded the highest values, with August–December values of 0.59, 0.80, 1.10, 1.30, and 1.40 mg 100 mL^−1^. In contrast, Shamber exhibited comparatively lower anthocyanin levels, including 0.04, 0.07, 0.09, 0.10, and 0.15 mg 100 mL^−1^ (Table 4).

**Figure 4:**
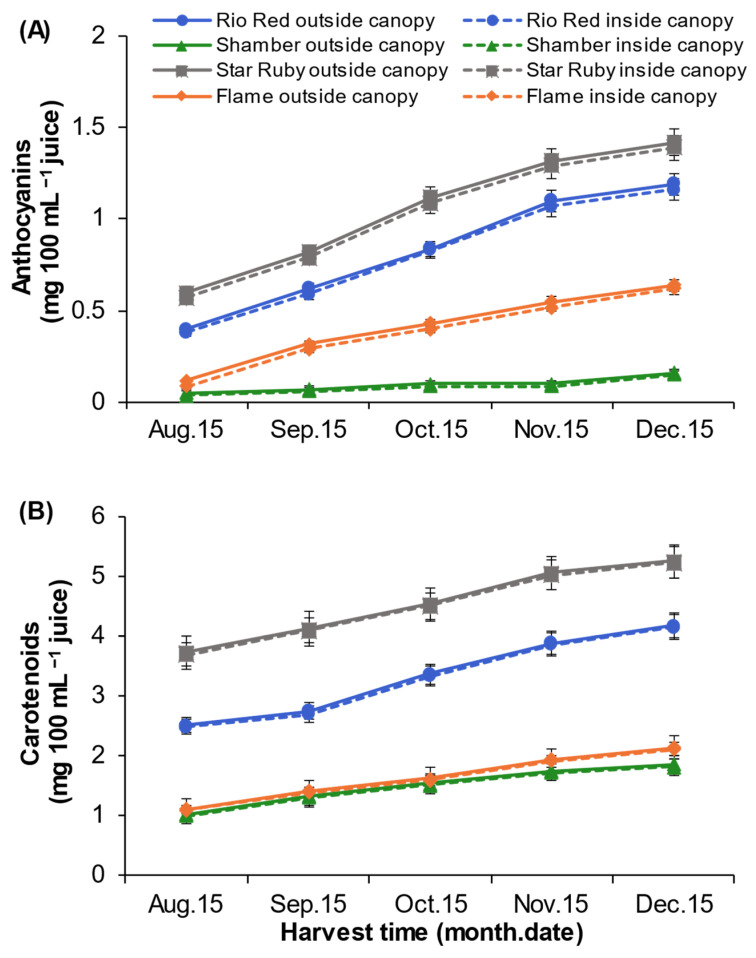
Changes in anthocyanins (**A**) and carotenoids (**B**) in the grapefruit cultivars, as affected by the cultivar and canopy positions at different harvest times (stages). The error bars represent the standard errors of the means (*n* = 3). The data for each harvesting stage were analyzed separately using two-way ANOVA (cultivar × canopy position), and the interaction means are presented graphically to show the stage-wise response of the cultivars across canopy positions during fruit development. Significance levels indicated as *p* < 0.01 (**), *p* < 0.05 (*), and NS = non-significant. CP × C represents canopy positions × cultivars. Anthocyanins CP × C: August–December^NS^. Carotenoids CP × C: August–December^NS^.

Regarding the interaction, the canopy position × cultivar was non-significant for both carotenoids and anthocyanins across all harvesting stages (August–December) (Figure 4A,B).

### 2.5. Pearson Correlation Matrix and Biplot Analysis

**Figure 5:**
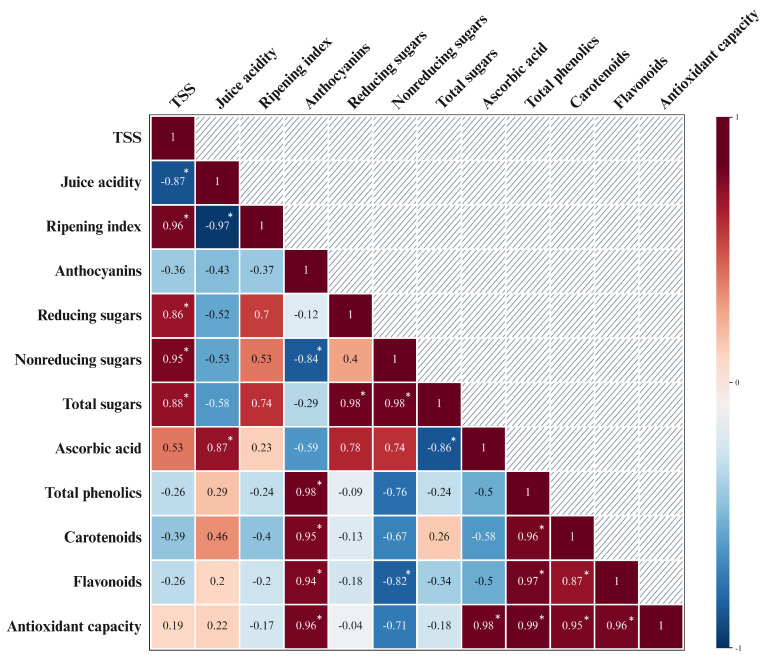
A heatmap correlation matrix representing correlations (Pearson coefficients) among the biochemical and antioxidant traits of the grapefruit cultivars harvested on 15 December (10 MAA). The values marked with an asterisk (*) are significant at *p* < 0.05 *.

For this purpose, only the data collected on 15 December were used because similar trends were observed across different maturity stages. TSSs were significantly positively correlated with reducing, nonreducing, and total sugars but negatively correlated with juice acidity. Juice acidity was significantly negatively associated with TSSs and the ripening index but positively correlated with ascorbic acid. The ripening index had a significant positive correlation with TSSs and a negative correlation with juice acidity. Anthocyanins were significantly negatively associated with nonreducing sugars but positively associated with total phenolic, carotenoid, flavonoid, and antioxidant capacity. Total sugars were significantly positively correlated with reducing and nonreducing sugars and negatively correlated with ascorbic acid. The antioxidant capacity was significantly positively correlated with the contents of anthocyanins, ascorbic acid, total phenolics, carotenoids, and flavonoids (Figure 5).

**Figure 6:**
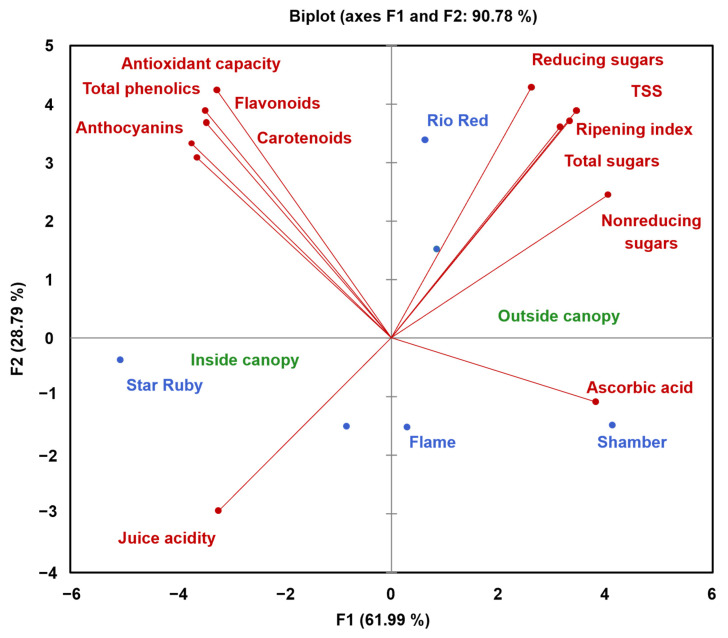
A PCA bioplot describing the cultivar (blue label) and canopy position (green label) effects on the segregation of grapefruit biochemical and antioxidant traits (red label) at harvest (15 December; ≈10 MAA).

Biplot analysis revealed considerable variation among the grapefruit cultivars and canopy positions that influenced fruit quality. The cultivar and canopy positions close to the trait vectors presented the maximum mean performance and a strong association with that trait. The Rio Red cultivar and outer canopy position were strongly linked with TSS, different sugars, and the ripening index. Star Ruby cultivar and inside canopy position were strongly associated with juice acidity. The Shamber cultivar was strongly associated with ascorbic acid. The Flame cultivar remained neutral and was not closely related to the other studied traits. However, the trait associations determined through biplot analysis were similar to those in the correlation matrix. The total phenolic content was significantly correlated with the anthocyanin, carotenoid, and flavonoid contents and the antioxidant capacity. Therefore, these traits and vectors were close to each other in the same quadrant (Figure 6).

## 3. Discussion

Biochemical and nutritional traits of grapefruit varied noticeably across canopy positions, cultivar, and stage of fruit development. In general, fruits from the outer canopy exhibited better fruit quality and nutritional profiles, with higher total soluble solids, ripening index, sugars, and pigment accumulation than those from the inner canopy. During fruit development, biochemical quality traits showed an increasing trend, while several bioactive compounds (total phenolic content, antioxidant capacity, and vitamin C) exhibited a decreasing trend towards maturity. Among the cultivars, Rio Red and Star Ruby attained more biochemical characteristics. Overall, fruit harvested in December represented a mature stage with balanced fruit quality and bioactive compounds.

Canopy position (outside and inside) creates microclimatic variation that influences the accumulation of biochemical metabolites and the quality of grapefruit cultivars (Table 1). In this study, outer canopy fruits consistently presented higher total soluble solids, ripening index, different sugars, and antioxidant metabolites than inner canopy fruits. These differences are likely associated with greater light exposure in the outer canopy, which may promote photosynthesis and assimilate allocation toward developing fruits, increasing sugar accumulation [8]. Inner canopy fruits are typically exposed to reduced light conditions within the canopy, which may influence carbohydrate accumulation and consequently affect TSSs levels. Optimal light exposure is necessary to produce high-quality fruits. Therefore, the present findings agree with previous reports showing improved fruit biochemical attributes under higher light availability [12]. Moreover, research on canopy architecture in peaches has demonstrated direct correlations between light intensity within canopy sections and the fruit sugar-to-acid ratio and color development [9], supporting the observed trends in the present study. Therefore, the current findings are in accordance with earlier work of several plant researchers. Overall, in the context of our study, higher biochemical attributes of the outer canopy may be related to greater light exposure within the canopy, which can increase photosynthetic activity and carbohydrate availability for developing fruits [8,13].

Outer canopy fruits presented significantly higher levels of anthocyanins, carotenoids, flavonoids, antioxidant capacity, phenolics, and ascorbic acid (Table 2), confirming the positive influence of light and temperature on pigment and antioxidant biosynthesis. Outer canopy fruits of blood oranges presented higher TSSs and anthocyanin concentrations and better color due to light exposure [14]. These metabolites are synthesized via light-dependent pathways; higher irradiance enhances phenylalanine ammonia lyase (PAL) and other key enzymes involved in flavonoid and carotenoid biosynthesis [14].

Consequently, fruits exposed to greater sunlight within the canopy may show slight increases in pigment accumulation and higher antioxidant potential, which can contribute to improved nutritional quality. In contrast, inner canopy fruits under shaded conditions may exhibit comparatively lower pigment accumulation and antioxidant activity [8]. This difference highlights how canopy management directly affects fruit nutritional composition and the potential health benefits of citrus juice. The maturity stage had a significant and progressive effect on fruit biochemical and antioxidant traits (Table 3). The quality of citrus fruits is defined by the stage of maturity/ripening of the fruit at the time of harvesting. The optimal harvest period is effective in terms of fruit quality and maximum yield [6]. In the present research, differences in the biochemical and bioactive compounds in the fruits were observed at various developmental/maturity stages from 15 August to 15 December, representing a continuous progression from early (≈6 MAA) to full physiological maturity (≈10 MAA), which offers a developmental structure applicable across regions with different flowering times.

Higher levels of antioxidants and pigments in fully matured fruits may result from prolonged light exposure, which stimulates the biosynthesis of anthocyanins and other phenolic compounds [8]. More recent studies have also confirmed that fruits harvested at later maturity stages under higher light conditions present enhanced carotenoid and phenolic profiles [15]. Similarly, Zhang et al. [16] reported higher TSSs and sugar levels and lower acidity levels in Gannan navel orange fruits.

The ripening index plays a significant role in determining the optimum fruit maturity and reducing quality loss. Fruits of superior quality, as estimated through TSSs, sugars, antioxidants, and bioactive compounds, can be obtained by determining the optimum harvesting time, and the decrease in organic acids was due to the ripening process of fruits, as indicated by Ahmed et al. [17], in grapefruits. Moreover, fruit sweetness is an integral part of quality and is directly associated with increased organic acid degradation and TSSs and sugar accumulation [16].

A decrease in ascorbic acid content, total phenolics, and antioxidant capacity with increasing maturity (15 August–15 December) was recorded in the studied grapefruit cultivars. Compared with unripe fruits, ripe fruits presented lower ascorbic acid contents, total phenolics, and antioxidant capacities, and a similar trend has been recorded by other researchers [17,18].

Climatic variables influence citrus fruit quality. Abrupt changes in climatic conditions result in poor yield and inferior fruit quality. Early or delayed fruit harvesting results in quality and yield losses of fruits due to immature and overripe fruits. Hence, timely harvesting of fruits is necessary for good fruit quality and productivity. From the present results, the optimum grapefruit harvesting time is December, which is similar to the findings of Singh et al. [19], who also concluded that December was the optimum harvesting time for grapefruit. Among the four cultivars studied, Rio Red and Star Ruby presented superior biochemical qualities characterized by higher TSSs, ripening index, sugars, phenolics, and antioxidant activities than those of Shamber and Flame. These findings suggest that cultivar genetics play a critical role in determining the biochemical potential and nutritional quality of grapefruit [11], particularly under variable canopy microclimates. Genotypic adaptability to environmental gradients, such as irradiance and temperature, could explain the observed variability among the cultivars [4]. Rio Red and Star Ruby resulted in greater pigment accumulation than Shamber and Flame, which is often associated with the presence of antioxidant compounds and improved nutritional attributes in citrus fruit. Collectively, these findings indicate that both canopy position and harvest maturity interact with cultivar genetics to define final fruit quality, suggesting that integrated orchard management strategies that combine canopy management, cultivar selection, and harvest scheduling can improve both fruit yield and biochemical and nutritional quality. The correlation matrix has the potential to suggest excellent perceptions for crop improvements. A positive correlation indicates that an enhancement in one trait significantly improves the other trait(s). Thus, multiple characteristics can be improved with the improvement of a single trait, which significantly contributes to the selection process for variety improvement and cultivar choice, as well [20]. Hence, a correlation matrix is an effective approach to optimize plant performance and ensure better fruit quality. In the present study, biplot analysis illustrates the impacts of grapefruit cultivar, canopy position, and maturity stage and their associations with fruit quality [6]. The Rio Red cultivar tends toward TSSs, different sugars, and ripening index, whereas the Star Ruby cultivar tends toward juice acidity, anthocyanins, flavonoids, and total phenolics. Moreover, the outside canopy presented greater ripening and sugar contents than the inside canopy. Correlation matrix and biplot analyses have been successfully applied to several crops, such as mandarins [6] and date palm [20].

## 4. Materials and Methods

### 4.1. Plant Materials and Experimental Site

In this study, healthy trees of four grapefruit cultivars (Rio Red, Shamber, Star Rubby and Flame) were selected from the Horticultural Research Station Sahiwal (30°39′ N, 73°06′ E; 173 m above sea level), Punjab, Pakistan, which experiences subtropical conditions (annual maximum temperature of 25–28 °C, minimum temperature of 18–22 °C, relative humidity of 64–69%, and rainfall of 210–275 mm). Light penetration and temperature variation were monitored between outer and inner canopy positions. All trees received uniform cultural and agronomic practices as recommended for commercial grapefruit production. Fruits were harvested from outer and inner canopy positions at five maturity stages (August–December) to assess the influence of canopy microclimate, harvest time, and cultivar differences in fruit biochemical composition, pigment accumulation, and quality-related physicochemical attributes. These maturity stages corresponded to ≈6–10 months after anthesis (MAA) on the basis of the observed flowering period under subtropical conditions.

### 4.2. Experimental Details

Harvested fruits were brought to the Department of Horticulture, Bahauddin Zakariya University, Multan (Pakistan), for biochemical analysis. The current study was arranged under a randomized complete block design (RCBD) with 3 replications and two experimental factors, canopy position and cultivar. The fruit positions on a tree were inside the canopy, i.e., fruit receiving less than 80% full sunlight, and the periphery of the canopy, i.e., fruit exposed to 90–100% full sunlight, according to Cronje et al. [21]. Four grapefruit cultivars, Rio Red, Shamber, Star Rubby, and Flame, were included in the study. For each replication, 8 fruits per canopy position were randomly harvested from 3 trees of each cultivar from a height of 1–2 m at different maturity stages. Fruit sampling was conducted monthly from 15 August to 15 December, when the fruits represented early (6 MAA) to full physiological maturity (10 MAA). The experiment was repeated for the second year using the same selected trees and other procedures.

### 4.3. TSSs (°Brix), Juice Acidity (%), and Ripening Index

A hand-held refractometer was used to determine the TSSs values of the fruit juice samples. A well-known procedure from Hortwitz [22] was used for the estimation of acidity levels in juice samples. The juice samples (10 mL) and distilled water (50 mL) were homogenized and titrated against 0.1 N NaOH solution until a light pink color appeared. Phenolphthalein (2–3 drops) was used for color indication.

The ripening index was calculated by using the following formula:

Ripening index = TSS/(juice acidity)

### 4.4. Estimation of Anthocyanin Content (mg 100 mL^−1^ Juice)

A solution of a fruit sample (2 mL) and methanol (18 mL) was kept for one hour for pigment removal, after which it was filtered through a Whatman No. 2 filter. After this, readings were recorded with a spectrophotometer (BMS, UV-1900/VS-1100) at a wavelength of 520 nm, as described by Borges et al. [23] with slight modification.

### 4.5. Sugars Estimation (%)

For the estimation of reducing, nonreducing, and total sugars, fruit juice (10 mL), lead acetate (25%, 25 mL), and potassium oxalate (20%, 10 mL) were homogenized, as previously described by Hortwitz [22]. The prepared filtrate was titrated against Fehling’s solution (10 mL) until the appearance of a brick-red color for the estimation of reducing sugars. Moreover, aliquots (25 mL), hydrochloric acid (HCl) (5 mL), and distilled water (20 mL) were homogenized and incubated for 48 h for the estimation of nonreducing sugars. Total sugars were calculated through the sum of reducing and nonreducing sugar levels in the fruit samples.

### 4.6. Ascorbic Acid Content (mg 100 mL^−1^ Juice)

Aliquots of juice samples (10 mL) and 0.4% oxalic acid (5 mL) were titrated against 2,6-dichlorophenolindophenol (dye), as described by Hortwitz [22]. The titration process continued until the appearance of a pink color.

### 4.7. Total Phenolics (mg GAE 100 mL^−1^ Juice)

Fruit juice (0.2 mL), Na_2_CO_3_ (7.5%, 0.8 mL), Folin–Ciocalteu reagent (5-fold diluted, 1 mL), and distilled water (10 mL) were homogenized and placed in darkness for approximately 30 min. The readings were subsequently recorded with a spectrophotometer (UV-3000, ORI, Brandenburg, Germany) at a wavelength of 765 nm [22].

### 4.8. Carotenoids (mg 100 mL^−1^ Juice)

The procedure of Lee and Castle [24] and Lang et al. [25], with slight modification, was used for the estimation of carotenoids. The fruit samples (2 mL) and acetone (5 mL) were mixed, and 25 mL of solution was mixed with distilled water in a volumetric flask. After filtration, readings were recorded with a spectrophotometer at a wavelength of 450 nm.

### 4.9. Flavonoids (mg QE 100 mL^−1^ Juice)

Juice samples (1 mL), sodium hydroxide (1 M, 2 mL), deionized water (4 mL), ethanol (5 mL), and sodium nitrite (300 µL, 5 g 100 mL^−1^) were homogenized and placed in a water bath (30 °C) for 60 min. After this, readings were recorded through a spectrophotometer at a wavelength of 510 nm, as described in [26,27].

### 4.10. Antioxidant Capacity (mM Trolox 100 mL^−1^ Juice)

A solution of a homogenized juice sample (1 mL) and DPPH (2,2-diphenyl-1-picrylhydrazyl) (1 mL) was prepared, and readings were recorded with a spectrophotometer at a wavelength of 571 nm [22,28].

### 4.11. Statistical Analysis

The data from the two research years did not show significant differences across all the measured traits and cultivars; the datasets were pooled and analyzed together (Table S1). The data were analyzed with Statistix 8.1 software (Tallahassee, FL, USA) via two-way (cultivars and canopy positions) analysis of variance (ANOVA) separately for each developmental/maturity stage. The treatment means were separated with the least significant difference (LSD) test at 0.05 probability. Pearson correlation matrix and biplot analysis were performed on the mean data collected on 15 December only, as this stage represented full fruit maturity, with peak expression of biochemical, pigment, and quality traits, allowing a precise assessment of their interrelationships. XLSTAT version 2025 was used for biplot analysis, and RStudio (version 4.3; R Foundation for Statistical Computing; Vienna Austria) was used for correlation matrix analysis.

## 5. Conclusions

The present study demonstrated that canopy microclimate, harvest maturity, and cultivar strongly influence the biochemical composition, phyto–nutritional quality, and antioxidant potential of grapefruit. Outer canopy fruits harvested in mid-December (≈10 MAA) exhibited peak sugar accumulation, optimum sweetness–acidity balance, and maximum level of bioactive metabolites. The cultivars Rio Red and Star Ruby outperformed Shamber and Flame, indicating better biochemical and nutraceutical attributes under subtropical orchard conditions. These findings highlight strategies for improving grapefruit health-promoting metabolite accumulation and nutritional quality through canopy management to improve light acceptance, high-quality cultivar selection, and maturity-based harvesting strategies. Overall, the results offer actionable guidance for growers, processors, and marketers to deliver fruit with superior nutritional and nutraceutical traits and commercial value. Future work should couple fruit quality assessments with canopy-specific concurrent leaf-level measurements. This will further clarify how photoassimilate production, transport, and distribution drive canopy microclimate effects on citrus fruit quality.

## Figures and Tables

**Table 1:** Effect of canopy position on juice characteristics and sugar content in grapefruit cultivars at different harvest times (stages) (August–December; 6 MAA–10 MAA).

Harvesting Times (Stages)	Canopy Positions	Juice Characteristics			Sugar Content		
		TSSs (°Brix)	Juice Acidity (%)	Ripening Index	Reducing Sugar (%)	Nonreducing Sugar (%)	Total Sugar (%)
August 15	Outside	6.64 a	2.58 b	2.61 a	3.16 a	1.47 a	4.63 a
	Inside	5.89 b	3.27 a	1.81 b	3.12 b	1.46 a	4.58 b
September 15	Outside	7.67 a	2.08 b	3.76 a	3.80 a	1.69 a	5.45 a
	Inside	6.89 b	2.76 a	2.51 b	3.73 b	1.64 b	5.43 a
October 15	Outside	7.98 a	1.07 b	7.53 a	4.50 a	2.10 a	6.60 a
	Inside	7.20 b	1.67 a	4.39 b	4.42 b	2.07 b	6.49 b
November 15	Outside	8.11 a	0.95 b	8.52 a	4.94 a	2.81 a	7.76 a
	Inside	7.33 b	1.18 a	6.34 b	4.89 b	2.76 b	7.65 b
December 15	Outside	8.13 a	0. 90 b	9.21 a	5.24 a	2.87 a	8.11 a
	Inside	7.35 b	1.15 a	6.50 b	5.18 b	2.82 b	8.00 b

All values presented are means of 3 replications; the different lowercase letters in each group indicate significant differences at *p* < 0.05 among the tested samples at outer and inner canopy positions, using the least significant difference test.

**Table 2:** Variation in juice characteristics and sugar content in grapefruit cultivars at different harvest times (stages) (August–December; 6 MAA–10 MAA).

Harvesting Times (Stages)	Cultivars	Juice Quality Attributes			Sugar Content		
		TSSs (°Brix)	Juice Acidity (%)	Ripening Index	Reducing Sugar (%)	Nonreducing Sugar (%)	Total Sugar (%)
August 15	Rio Red	6.90 a	2.80 a	2.62 a	4.00 a	1.47 b	5.47 a
	Shamber	6.58 a	2.90 a	2.30 b	3.29 b	1.50 ab	4.79 b
	Star Ruby	5.41 c	3.00 a	1.84 d	2.46 d	1.52 a	3.98 d
	Flame	6.18 b	3.00 a	2.10 c	2.81 c	1.38 c	4.18 c
September 15	Rio Red	7.94 a	2.30 a	3.76 a	4.46 a	1.99 a	6.46 a
	Shamber	7.57 b	2.40 a	3.21 b	4.27 b	1.52 c	5.79 b
	Star Ruby	6.41 d	2.50 a	2.63 d	2.94 d	1.52 c	4.47 d
	Flame	7.18 c	2.50 a	2.94 c	3.40 c	1.64 b	5.05 c
October 15	Rio Red	8.25 a	1.38 a	6.66 a	5.45 a	2.22 a	7.67 a
	Shamber	7.88 b	1.30 a	6.37 ab	4.79 b	2.00 c	6.79 b
	Star Ruby	6.72 d	1.40 a	5.12 c	3.62 d	1.95 d	5.57 d
	Flame	7.49 c	1.40 a	5.70 bc	3.99 c	2.16 b	6.15 c
November 15	Rio Red	8.38 a	1.01 b	8.37 a	5.87 a	2.89 b	8.77 a
	Shamber	8.01 b	1.01 b	8.01 a	5.08 b	3.02 a	8.10 b
	Star Ruby	6.85 d	1.15 a	6.16 c	4.33 d	2.44 d	6.77 d
	Flame	7.62 c	1.09 ab	7.18 b	4.38 c	2.80 c	7.19 c
December 15	Rio Red	8.42 a	0.92 b	9.53 a	6.17 a	2.81 b	8.99 a
	Shamber	8.03 b	0.99 b	8.21 b	5.39 b	3.11 a	8.50 b
	Star Ruby	6.87 d	1.13 a	6.20 d	4.44 d	2.69 d	7.14 d
	Flame	7.64 c	1.05 ab	7.46 c	4.83 c	2.76 c	7.59 c

All values presented are means of 3 replications; the different lowercase letters in each group indicate significant differences at *p* < 0.05 among the tested samples for each cultivar, using the least significant difference test.

**Table 3:** Effect of canopy position on bioactive compounds and pigment content in grapefruit cultivars at different harvest times (stages) (August–December; 6 MAA–10 MAA).

Harvesting Times (Stages)	Canopy Positions	Bioactive Compounds			Pigment Content		
		Ascorbic Acid (mg 100 mL^−1^ Juice)	Total Phenolics (mg GAE 100 mL^−1^ Juice)	Antioxidant Capacity (mM Trolox 100 mL^−1^ Juice)	Flavonoids (mg QE 100 mL^−1^ Juice)	Carotenoids (mg 100 mL^−1^ Juice)	Anthocyanins (mg 100 mL^−1^ Juice)
August 15	Outside	59.62 a	84.63 a	36.36 a	15.57 a	2.09 a	0.29 a
	Inside	57.10 b	82.69 b	34.38 b	13.59 b	2.06 b	0.27 b
September 15	Outside	57.62 a	82.62 a	34.11 a	20.33 a	2.40 a	0.46 a
	Inside	55.10 b	80.68 b	32.15 b	20.42 a	2.36 b	0.43 b
October 15	Outside	57.62 a	80.14 a	30.49 a	26.61 a	2.76 a	0.62 a
	Inside	55.10 b	78.20 b	28.54 b	24.62 b	2.73 b	0.60 b
November 15	Outside	55.12 a	78.60 a	28.37 a	29.81 a	3.15 a	0.77 a
	Inside	52.60 b	76.66 b	26.41 b	27.83 b	3.12 b	0.74 b
December 15	Outside	54.87 a	78.27 a	27.47 a	31.33 a	3.35 a	0.85 a
	Inside	52.35 b	76.33 b	25.51 b	29.34 b	3.32 b	0.83 b

All values presented are the means of 3 replications; the different lowercase letters in each group indicate significant differences at *p* < 0.05 among the tested samples at outer and inner canopy positions, using the least significant difference test.

**Table 4:** Variation in bioactive compounds and pigment content in grapefruit cultivars at different harvest times (stages) (August–December; 6 MAA–10 MAA).

Harvesting Times (Stages)	Cultivars	Bioactive Compounds			Pigment Content		
		Ascorbic Acid (mg 100 mL^−1^ Juice)	Total Phenolics (mg GAE 100 mL^−1^ Juice)	Antioxidant Capacity (mM Trolox 100 mL^−1^ Juice)	Flavonoids (mg QE 100 mL^−1^ Juice)	Carotenoids (mg 100 mL^−1^ Juice)	Anthocyanins (mg 100 mL^−1^ Juice)
August 15	Rio Red	60.00 a	86.45 a	37.18 b	15.04 b	2.50 b	0.38 b
	Shamber	60.46 a	79.16 c	32.13 d	13.24 d	1.01 d	0.04 d
	Star Ruby	55.00 c	86.82 a	38.12 a	15.68 a	3.70 a	0.59 a
	Flame	58.00 b	82.22 b	34.05 c	14.34 c	1.09 c	0.10 c
September 15	Rio Red	58.25 a	84.20 b	35.03 b	21.09 b	2.71 b	0.60 b
	Shamber	58.46 a	77.17 d	30.00 d	18.24 d	1.31 d	0.07 d
	Star Ruby	53.25 c	85.07 a	35.82 a	21.83 a	4.10 a	0.80 a
	Flame	56.00 b	80.17 c	31.65 c	20.34 c	1.40 c	0.30 c
October 15	Rio Red	58.00 a	81.50 b	31.69 b	27.59 b	3.34 b	0.83 b
	Shamber	58.25 a	74.62 d	25.90 d	22.04 d	1.52 d	0.09 d
	Star Ruby	53.00 c	83.17 a	32.82 a	27.98 a	4.52 a	1.10 a
	Flame	56.00 b	77.39 c	27.65 c	24.84 c	1.60 c	0.41 c
November 15	Rio Red	55.50 a	80.00 b	29.69 b	29.59 b	3.86 b	1.08 b
	Shamber	55.96 a	73.46 d	23.90 d	25.02 d	1.73 d	0.10 d
	Star Ruby	50.50 c	81.67 a	30.32 a	31.83 a	5.04 a	1.30 a
	Flame	53.50 b	75.39 c	25.65 c	28.84 c	1.92 c	0.53 c
December 15	Rio Red	55.25 a	79.85 b	28.69 b	31.59 b	4.15 b	1.17 b
	Shamber	55.71 a	72.50 d	22.90 d	27.02 d	1.83 d	0.15 d
	Star Ruby	50.25 c	81.61 a	29.73 a	32.88 a	5.24 a	1.40 a
	Flame	53.25 b	75.24 c	24.65 c	29.84 c	2.12 c	0.63 c

All values presented are the means of 3 replications; the different lowercase letters in each group indicate significant differences at *p* < 0.05 among the tested samples for each cultivar, using the least significant difference test.

## Data Availability

The original contributions presented in this study are included in the article. Further inquiries can be directed to the corresponding author.

## Supplementary Materials

The following supporting information can be downloaded at: https://www.mdpi.com/article/10.3390/plants15111750/s1, Table S1: Data from the second year of the study for all measured variables.
